# Radiation-free mother-baby endoscopic balloon dilation for cervical esophago-ileal anastomotic stenosis

**DOI:** 10.1055/a-2512-3975

**Published:** 2025-02-11

**Authors:** Su-Yu Chen, De-Rong Zhang, Hong Shi

**Affiliations:** 1 Endoscopy Center, Clinical Oncology School of Fujian Medical University, Fujian Cancer Hospital, Fujian Branch of Fudan University Shanghai Cancer Center, Fuzhou, China; 2 Thoracic Surgery, Clinical Oncology School of Fujian Medical University, Fujian Cancer Hospital, Fujian Branch of Fudan University Shanghai Cancer Center, Fuzhou, China

A 68-year-old man presented to our hospital with progressive dysphagia and retrosternal discomfort for 1 month. Endoscopy and pathological biopsy confirmed invasive squamous cell carcinoma of the distal esophagus ( Fig. 1 **a** ) and poorly differentiated adenocarcinoma at the junction of the gastric antrum and body ( Fig. 1 **b** ). Following preoperative preparation, the patient underwent subtotal esophagectomy, gastrectomy, and anterograde ileo-colon interposition for esophageal replacement via the retrosternal route.

**Fig. 1:**
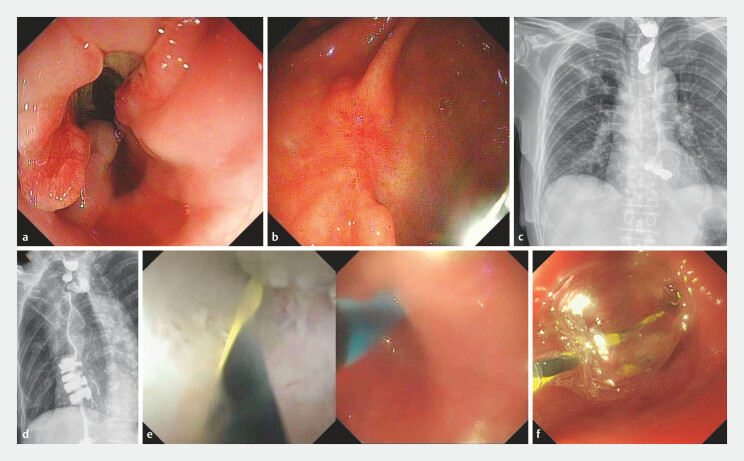
**a** Endoscopic image of invasive squamous cell carcinoma in the distal esophagus. **b** Poorly differentiated adenocarcinoma at the junction of the gastric antrum and body. **c–d** Esophagogram revealed tortuous angulation of the cervical esophago-ileal anastomosis. **e** Intestinal villi identified by intraluminal saline irrigation. **f** Endoscopic balloon dilation.

On postoperative day 44, an esophagogram revealed tortuous angulation at the cervical esophago-ileal anastomosis ( Fig. 1 **c, d** ). On postoperative day 65, the patient started experiencing difficulty swallowing water due to an anastomotic stenosis with a diameter less than 5 mm, as revealed by endoscopy. The initial attempt at endoscopic balloon dilation failed because the guidewire could not be advanced under direct visualization.

Subsequently, radiation-free mother-baby endoscopic balloon dilation using a TY-ISS-L31 catheter (outer diameter: 3.1 mm; Vedkang, Changzhou, China) was planned ( Video 1 ). The catheter was introduced through the working channel of a therapeutic endoscope (GIF-1T240; inner channel diameter: 4.2 mm; Olympus, Tokyo, Japan) and positioned at the anastomotic site. The catheter was then advanced slowly into the ileal lumen until the guidewire reached the cecum. Intestinal villi were visualized during insertion using intraluminal saline irrigation ( Fig. 1 **e** ).

Radiation-free mother-baby endoscopic balloon dilation performed for cervical esophago-ileal anastomotic stenosis in a 68-year-old man.Video 1

The TY-ISS-L31 catheter was subsequently withdrawn, leaving the guidewire in place. A balloon (12–13.5–15 mm; Micro-Tech, Nanjing, China) was inserted over the guidewire to dilate the cervical anastomosis ( Fig. 1 **f** ). Post-procedure, the conventional endoscope passed through the cervical anastomosis and ileocecal valve without resistance.

This report highlights the first use of a mother-baby endoscopic technique, without fluoroscopic guidance, to dilate an angulated esophago-ileal anastomosis. This balloon dilation approach exclusively using endoscopy avoids radiation exposure for both patients and medical personnel, making it particularly beneficial for radiation-intolerant patients or institutions lacking fluoroscopic equipment. This novel technique expands the possibilities for radiation-free endoscopic procedures in confined anatomical spaces 1 2 3 .

Endoscopy_UCTN_Code_CCL_1AB_2AC_3AD

## Notes

Endoscopy E-Videos https://eref.thieme.de/e-videos
*E-Videos* is an open access online section of the journal *Endoscopy* , reporting on interesting cases and new techniques in gastroenterological endoscopy. All papers include a high-quality video and are published with a Creative Commons CC-BY license. Endoscopy E-Videos qualify for HINARI discounts and waivers and eligibility is automatically checked during the submission process. We grant 100% waivers to articles whose corresponding authors are based in Group A countries and 50% waivers to those who are based in Group B countries as classified by Research4Life (see: https://www.research4life.org/access/eligibility/ ). This section has its own submission website at https://mc.manuscriptcentral.com/e-videos .

## References

[1] Tang L Zhao H Peng K Use of biliopancreatic duct imaging system in a 13-month-old girl to enable endoscopic removal of a foreign body in the appendix Endoscopy 2024 56 E408 E409 10.1055/a-2308-2975 38759964 PMC11101268

[2] Kong LJ Liu D Zhang JY Digital single-operator cholangioscope for endoscopic retrograde appendicitis therapy Endoscopy 2022 54 396 400 10.1055/a-1490-0434 33893629

[3] Laleman W Verraes K Van Steenbergen W Usefulness of the single-operator cholangioscopy system SpyGlass in biliary disease: a single-center prospective cohort study and aggregated review Surg Endosc 2017 31 2223 2232 10.1007/s00464-016-5221-2 27604370

